# The clock gene PER2 and sleep problems: Association with alcohol consumption among Swedish adolescents

**DOI:** 10.3109/03009731003597127

**Published:** 2010-03-10

**Authors:** Erika Comasco, Niklas Nordquist, Camilla Göktürk, Cecilia Åslund, Jarmila Hallman, Lars Oreland, Kent W. Nilsson

**Affiliations:** ^1^Centre for Clinical Research, Uppsala University, Central Hospital, Västerås 721 89 VästeråsSweden; ^2^Department of Neuroscience, Unit of Pharmacology, Uppsala University, BMC, Box 593, 751 24 UppsalaSweden; ^3^Department of Neuroscience, Unit of Psychiatry, Uppsala University Hospital, 751 85 UppsalaSweden

**Keywords:** Adolescents, alcohol, AUDIT, clock gene, PER2, sleep problems

## Abstract

**Background:**

Alcohol abuse is associated with sleep problems, which are often linked to circadian rhythm disturbances. Previous studies have separately examined the effects of mutations in the clock gene *PER2* on alcohol consumption and sleep problems. Here we hypothesized that an allelic variation in the *PER2* gene is associated with alcohol consumption in interaction with sleep problems among adolescents.

**Methods:**

The Survey of Adolescent Life and Health in Västmanland 2006, a Swedish county, including 1254 students 17–18 years old, was used as a population-representative sample of adolescents. We investigated the *PER2* Single Nucleotide polymorphism (SNP) 10870 (A/G) in the cohort together with an assessment of alcohol consumption according to the AUDIT-C questionnaire, and sleep problems using a survey consisting of 18 items. Furthermore, we carried out an exploratory analysis on the *PER2* Single Nucleotide Polymorphism 10870 polymorphism in a group of severely alcoholic females.

**Results:**

We found a significant association of the SNP 10870 in adolescent boys, where the genotype AA, in the presence of several and frequent sleep problems, was associated with increased alcohol consumption. Among adolescent girls, only sleep problems were related to alcohol consumption. A non-significant trend was observed among the severely alcoholic females, with the G allele being over-represented in the severely alcoholic females group in comparision to the control females.

**Conclusion:**

These results indicate that *PER2* gene variation is associated with alcohol consumption in interaction with sleep problems among Swedish adolescent boys.

## Introduction

Alcohol is implicated in a wide variety of diseases and injuries, as well as in many social and legal problems (1). Adolescent drinking is a topic of public concern (2) and has been linked to a range of psycho-social problems, which can result in negative consequences lasting well into adulthood (3). Alcoholism is often co-morbid with sleep problems (4).

Adequate sleep provides a crucial restorative function necessary for the control of attention, emotion, and behaviour. Moreover, complaints about sleep quality are common among adolescents (5). It is not surprising, therefore, that research has consistently linked sleep problems to emotional and behavioural problems in adolescents, including both internalizing and externalizing disorders (5,6). Sleep problems have been defined as any subjectively perceived or objectively measured problem with sleep (4). However, it has been shown that quantitative laboratory measures of sleep quality are not always correlated with subjective perception of sleep, such as ‘depth’ or ‘restfulness’ of sleep upon awakening (7).

Sleep problems are common among alcoholics (4), but relatively little is known about the relationship between sleep problems and alcohol use in adolescence (8). Adolescence is a particularly interesting developmental period because of changes in both sleep patterns (9,10) and alcohol use (2). Alcohol use and abuse often begin to develop during adolescence. Thus, examination of sleep problems during this developmental period may be valuable as a potential risk indicator of early onset of alcohol abuse. On the other hand, alcohol abuse during adolescence might disrupt circadian rhythmicity and therefore be associated with sleeping problems (8).

Disruption of normal circadian rhythmicity has been associated with various psychiatric diseases, including sleep disorders and alcoholism (11,12). Altered circadian rhythm as a biological response to ethanol has been observed in mice (13). However, only recently the focus has shifted to the chronobiological effects of alcohol as a direct moderator of the circadian pace-maker (14). Chen et al. described an alteration in the circadian rhythm in male rats caused by chronic ethanol consumption, identifying the *Drosophila* homologue period gene *PER2* as a potential mediator (15). *PER2* gene participates in regulating the transcription of other clock genes and regulates its own transcription through a gene–protein–gene feedback loop in the suprachiasmatic nucleus (SCN) of the anterior hypothalamus, which is the site of the principal circadian clock of mammals (16–18). Studies on rodents have reported mutations in the *PER2* gene that influence the mammalian circadian clock (19,20). Additional studies have shown an influence of *PER2* on behavioural traits, such as cocaine-induced locomotor sensitization and reward in mice (21) and in *Drosophila* (22). In humans, a mutation in the *PER2* gene has been shown to cause the familial advanced sleep phase syndrome (FASPS) (23). The *PER2* has also been associated with seasonal affective disorders (24).

Furthermore, a recent study suggested *PER2* as a candidate gene for alcohol consumption in both rodents and humans. Spanagel et al*.* demonstrated that PER2^Brdm1^ mutant mice, in which the *PER2* gene was disrupted, exhibited enhanced alcohol intake and preference compared to wild-type mice. A significant association was also shown, in a clinical sample of severe alcoholics, between the *PER2* SNP 10870 and alcohol intake (25). The *PER2* SNP 10870, an A/G substitution, is located in an enhancer-like structure in intron 3 of chromosome 2, containing potential transcription factor binding motifs which have been found to be altered by this SNP and thus having a possible regulatory function in the transcriptional activation of the *PER2* gene (24,25).

In conclusion, alcohol abuse has been associated with sleep problems, which are often linked to circadian rhythm disturbances. In this study we have hypothesized that the genetic variation SNP 10870 in the *PER2* gene might affect alcohol consumption directly or in interaction with sleep problems. This hypothesis was tested in a population representative sample of adolescents. Furthermore, we carried out an exploratory analysis on the *PER2* SNP 10870 polymorphism in a group of severely alcoholic females.

## Materials and methods

### Participants

#### Adolescents

The present study was part of the Survey of Adolescent Life and Health in Västmanland 2006 (SALVè 2006), a survey biennially distributed by the County Council of Västmanland, Sweden, in order to monitor the psycho-social health of the adolescent population of the county. Västmanland is a medium-sized county in Sweden, about 100 kilometres north-west of Stockholm. According to Statistics Sweden, in Västmanland, in the year 2005, 91% of the adolescent population went to the non-compulsory secondary school from primary school, 4.3% went to other types of schools, and the rest were out of school (26). All students in second year of secondary school (17–18 years old), comprising 87% of the population for this age group, were asked to complete a questionnaire during class hours. Questionnaires were left with the teacher to be given to the students not attending class at the time of the study. In addition, participants were asked to provide a saliva sample for DNA extraction by rinsing their mouth for 30 seconds with an isotonic salt solution. Participation was anonymous and voluntary. A total of 2468 students completed the questionnaire (77.4% of the target population with a 97.7% internal response rate), of whom 183 late-respondents returned their questionnaires by mail. A saliva sample was provided by 2131 participants. Only adolescents who stated in the self-report to be born in Sweden and to have Swedish parents were included in the study. Due to problems with DNA isolation, genotype analyses, or missing answers to the questions, the final study sample comprised 660 boys and 594 girls. The study was approved by the regional ethical review board of Uppsala University.

#### Severely alcoholic females

A sample of 110 Caucasian in-patients was recruited between July 2001 and July 2006 from a long-term in-patient treatment facility for females suffering from alcoholism and drug abuse (27). Most patients treated were sent to the facility by social authorities (107 out of the 110 were treated in accordance to a court order). A physician and a specialist in psychiatry examined all patients; data were collected from interviews and clinical records. All in-patients were included in the study after having fulfilled the diagnostic criteria for alcohol dependence according to ICD-10 Diagnostic Criteria for Research (28). Alcoholics who had been diagnosed with one or more psychiatric diseases were excluded. The age of the remaining patients (*n* = 58) ranged from 18 to 71 years. Blood samples for genetic analyses were collected from the patients. The study was approved by the regional ethical review board of Uppsala University.

### Control females. 

The adolescent females (n=594) part of the 2006 survey of adolescent life in Västmanland (SALVé 2006) were used as control group.

### Procedures

#### PER2 genotyping

The *PER2* gene SNP 10870 polymorphism was amplified from 3–20 ng genomic DNA using a Custom TaqMan^®^ SNP Genotyping Assay (Applied Biosystems), which contains sequence-specific forward and reverse primers and two TaqMan^®^ MGB probes labelled with a FAM^TM^ or VIC^®^ reporter dye at the 5′ end and a non-fluorescent quencher at the 3′ end. PCR primers were designed on the basis of the PER2 sequence published at www.ensemble.org. PCR was performed in a 5 μL reaction mixture containing TaqMan^®^ Universal PCR Master Mix (Applied Biosystems) 2.5 μL; 40 × Custom TaqMan^®^ SNP Genotyping Assays Mix (Applied Biosystems) 0.125 μL, and 3–20 ng genomic DNA diluted in H_2_O. The allele discrimination polymerase chain reaction was performed on an ABI PRISM^®^ 7900HT sequence detection system at the following thermal cycler conditions: initial step of 10 min at 95°C, followed by 40 cycles of denaturation 15 sec 92°C and annealing 60 sec 60°C. Genotypes were analysed using SDS 2.3 (Applied Biosystems^®^). To estimate the rate of genotyping errors, one quarter of the sample was genotyped and analysed twice; the comparison indicated no inconsistencies.

#### Assessment of sleep problems

Sleep complaints were recorded based on the Karolinska Sleep Questionnaire (29), comprising 18 questions, inquiring about frequency of sleep disturbances and subjective sleep quality. ‘During the last three months, how often have you experienced: difficulties falling asleep; difficulties awaking up; repeated awakenings with difficulty going back to sleep; heavy snoring (according to surroundings); insufficient sleep; light and superficial sleep; breathing interruptions during sleep (according to surroundings); nightmares; not thoroughly rested; premature (final) awakening; disturbed sleep; feeling to be exhausted at awakening; sleepiness during school time/work; sleepiness during spare time; drowsiness/prolonged fatigue; involuntary naps (till late supper) during school time or work; involuntary naps (till late supper) during spare time; having to fight sleep to be able to stay awake’. The possible answers were scored as: 1: never; 2: seldom, occasional moments; 3: sometimes, few times per month; 4: often, 1–2 times per week; 5: mostly, 3–4 times a week; 6: almost always, 5 times per week or more.

The component score has been summed to produce a ‘sleep problem index’ (range of 1–108). Individuals who scored >44 (sleep problem index mean = 44), have been considered having ‘several and frequent sleep problems’ (37.9% boys and 54.5% girls).

#### Assessment of alcohol consumption

The participants (SALVè 2006) answered the first three questions of the Alcohol Use Disorders Identification Test (AUDIT) to measure risk consumption, AUDIT-C (30). The three questions were: 1) How often did you have a drink containing alcohol in the last 12 months? 2) How many drinks containing alcohol do you have on a typical day when you are drinking? 3) How often do you have six or more drinks on one occasion? The scores of the three items have been summed to produce the ‘AUDIT-C index’ (range of 0–12). A cut-off point of 5 was used to differentiate subjects with regard to high alcohol consumption. This dichotomous (0–5 versus 6–12) measure of heavy drinking has been shown to have sensitivity of 0.82 and a specificity of 0.67 among older adolescents (31).

### Statistical analyses

Pearson's chi-square test was used to determine group differences on dichotomous measures. The non-parametric Kruskal-Wallis test was used for univariate analyses, and Spearman's rho test for bivariate correlation test between AUDIT-C and sleep problems indexes. The Kolmogorov-Smirnov (K-S) test was used to test for normal distribution. In a general linear model (GLM) univariate analysis, the *PER2* polymorphism and sex have been used as independent variables, sleep problems index as a covariate, and AUDIT-C index as the dependent variable. In a binary logistic regression analysis dichotomous measures were entered for the *PER2* polymorphism, sleep problems and the interaction term between the two as independent variables, and alcohol consumption as dependent variable.

## Results

The mean of the AUDIT-C index was 4.5 (SD 3.1) in boys and 3.8 (SD 2.5) in girls; the mean of the sleep problems index was 41.6 (SD 13.3) in boys and 47.1 (SD 14.3) in girls.

Girls experienced sleep problems significantly more often than did boys (chi-square = 34.99, *df* = 1, *P* <0.001), while boys reported significantly higher alcohol consumption than girls (x^2^ = 41.43, df = 1, p < 0.001).

The genotypes for the SNP 10870 in the clock gene *PER2* have been analysed as two groups, homozygosity for the A allele versus presence of at least one G allele, due to the small frequency of the G allele (Table I).

**Table I.** Genotype and the allele frequencies of the *PER2* SNP 10870 among Swedish adolescents.

*PER2* SNP 10870	Total	Boys	Girls
A allele	0.87	0.87	0.87
G allele	0.13	0.13	0.13
AA	958 (76.4%)	506 (76.7%)	452 (76.1%)
AG	269 (21.5%)	140 (21.2%)	129 (21.7%)
GG	27 (2.2%)	14 (2.1%)	13 (2.2%)
Total *n*	1254 (100%)	660 (100%)	594 (100%)
Hardy Weinberg equilibrium	Chi-square = 2.43 *P* = 0.12	Chi-square = 1.34 *P* = 0.25	Chi-square = 1.01 *P* = 0.30

**Figure 1.**
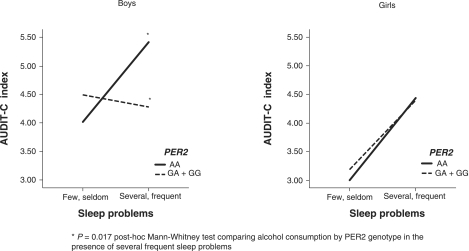
Line-chart describing the relationship between alcohol consumption and sleep problems by *PER2* SNP 10870 genotypes among Swedish adolescents.

In a non-parametric univariate analysis, the *PER2* 10870 polymorphism did not show any association neither with sleep problems (chi-square = 0.67, *df* = 1, *P* = 0.41 in boys; chi-square = 1.55, *df* = 1, *P* = 0.21 in girls) nor with alcohol consumption (chi-square = 0.30, *df* = 1, *P* = 0.58 in boys; chi-square = 0.001, *df* = 1, *P* = 0.97 in girls). Alcohol consumption was significantly correlated positively to sleep problems in boys (*r* = 15.43, *P* <0.001) and in girls (*r* = 29.78, *P* <0.001). However, in a multivariate analysis using a general linear model (Table II), adjusted for direct effects as well as an interaction effect, the *PER2* was directly related to alcohol consumption and interacted with sleep problems in relation to alcohol consumption (Adj. *R^2^* = 0.07). Among boys, the main and interaction effect of the *PER2* genotype had stronger impact on alcohol consumption than sleeping problems (Adj. *R^2^* = 0.06). Among girls, the *PER2* genotype had no main or interaction effect in relation to alcohol consumption, whereas sleep problems were significantly related to alcohol consumption (Adj. *R^2^* = 0.07) (Table II). Among boys the *PER2* AA genotype was associated with sleep problems and high alcohol consumption (Figure 1). The directions of the genotype effect and sleep problems on alcohol consumption are shown in Figure 1.

**Table II.** General linear model of direct- and interaction effects of *PER2* SNP 10870 genotype and sleep problems in relation to alcohol consumption among Swedish adoloscents with *df* degrees of freedom, *F*-test and *p* significance values

Alcohol consumption (AUDIT-C index)									
	Total (n = 1254)			Boys (*n* = 660)			Girls (*n* = 594)		
	*df*	*F*	*P*	*df*	*F*	*P*	*df*	*F*	*P*
*PER2*	1	5.83	0.016	1	15.16	<0.001	1	0.75	0.388
Sleep problems index	1	36.37	<0.001	1	8.03	0.005	1	41.64	<0.001
*PER2* × Sleep problems	1	6.63	0.010	1	18.49	<0.001	1	1.18	0.278
Sex	1	39.88	<0.001	-	-	-	-	-	-
Explained variance	Adj. *R^2^* = 0.071			Adj. *R^2^* = 0.064			Adj. *R^2^* = 0.073		

A non-parametric method was also used to re-test our findings from the GLM, since neither the AUDIT-C nor the sleep problem indexes were normally distributed. The results were confirmed by the binary logistic regression analysis. We found a significant interaction between the *PER2* and sleep problems in boys (*B* = 0.92; Wald = 5.5; *df =*1; *P* = 0.02; OR = 2.51; 95% CI 1.16–5.41) but not among girls. In a qualitative analysis, comparing the *PER2* genotype frequencies between adolescents who never drank and adolescents who drank, there was no significant difference (chi-square = 0.21; *P* = 0.65 in boys; chi-square = 0.45; *P* = 0.50 in girls), when adolescents who never drank (15.8%) were excluded from the model, the GLM univariate analysis confirmed the significant interaction effect between the *PER2* and sleep problems among boys (*F* = 3.97; *df* = 1; *P* = 0.05; Adj. *R^2^* = 0.05).

Additionally, we investigated the *PER2* SNP 10870 in a group of severely alcoholic females. The genotype frequency was in Hardy Weinberg equilibrium (chi-square = 2.52, *P* = 0.11 among alcoholics, and chi-square = 1.02, *P* = 0.31 among the controls). The G allele frequency was 0.17 among alcoholics and 0.13 among the control females. A Pearson's chi-square test was used to compare the *PER2* genotype frequencies among the alcoholic females group versus the control females (GG + GA versus AA). The results showed a non-significant trend (chi-square = 3.17, *df* = 1, *P* = 0.07), with the G allele being over-represented in the alcoholic females group in comparison to the control females sample (Table III).

**Table III.** Comparision of the genotype ditribution of the *PER2* SNP 10870 between severely alcoholic females and control females

		Genotype distribution		
*PER2* SNP 10870	*n*	AA	AG	GG
Alcoholic females	58	38 (65.5%)	20 (34.5%)	0 (0.0%)
Control females	594	452 (76.1%)	129 (21.7%)	13 (2.2%)
Pearson chi-square test^a^		X^2^ = 3.17, *df* = 1, *P* = 0.07		

^a^Chi-square test comparing the genotypic distribution frequencies (GG or GA vs. AA) between cases and controls.

## Discussion

The first part of the present study aimed at highlighting the potential role of variation in the clock gene *PER2* and sleep problems on alcohol consumption among a population representative sample of adolescents. The novelty of this study relies on the absence of studies in the literature of such a combination.

Our findings support an association between alcohol consumption in adolescents and variation in the *PER2* gene in an interaction with sleep problems. We found a significant association with the SNP 10870 in adolescent boys, in whom the AA genotype, in the presence of several and frequent sleep problems, was associated with increased alcohol consumption. Among adolescent girls, only sleep problems were associated with alcohol consumption, and no gene effect was present. Among the severely alcoholic females group, a non-significant trend was observed, with the G allele being over-represented in comparison to the control female sample.

Taken together, these results suggest the G allele as a protective factor for high alcohol consumption in boys, which is in accordance with the study by Spanagel et al*.* (25), since it included individuals of whom males were in majority (76.9%) (32).

The link between alcohol consumption and circadian rhythmicity has gained increasing interest in recent years (33); circadian abnormalities and sleep problems have been related to psychological distress, including anxiety and depression (24,34,35). The chronobiological effects of alcohol could potentially be mediated in part by the neurotransmitter glutamate, since ethanol's interactions with the glutamatergic neurotransmitter pathways, especially at the level of the N-methyl-D-aspartate receptor, have been suggested to be involved in alcohol's effects (36). Glutamate seems to be a regulator of the circadian clock system through its release at the synapses of the retinal projections to the SCN (37). The presence of a hyperglutamatergic state due to a down-regulation of the glutamate transporter GLAST in PER2 mutant mice has been shown to contribute to increased alcohol consumption (25). Additionally the effectiveness of acamprosate, a drug used to reduce alcohol consumption and risk of relapse, was observed to be higher in PER2 mutant mice (25).

A previous study has shown that PER2 mutant mice have altered monoamine oxidase A expression and dopaminergic activity (38), thus suggesting that neurotransmitter systems involved in mood-related behaviours, such as the dopaminergic, glutamatergic, serotonergic, noradrenergic, and GABAergic systems, could be modulated by the circadian system.

The high correlation rate between sleep problems and alcohol abuse is likely to be bidirectional. Because alcohol has a known sedative effect, it is plausible that some individuals with sleep disturbances will use alcohol to self-medicate their sleep problems with the possibility that sleep problems may play a causal role in the onset of alcohol problems. On the other hand, it is also plausible that excessive alcohol, i.e. binge drinking and drunkenness, contributes directly to disrupting circadian rhythmicity and consequently to the development of sleep problems. A vicious cycle can be initiated, and the combination of alcohol tolerance and sleep disruption can lead to an escalation of alcohol intake and subsequent alcohol-related problems. A longitudinal approach would help to better understand the casual-consequential relationship between sleep and alcohol problems.

Among the severely alcoholic females, our results suggest the *PER2* G allele as a possible risk factor for alcoholism. Among adolescent girls, only an association between sleep problems and alcohol consumption was observed. Moreover, a recent study reported the *PER2* G allele as a risk variant for winter depression in a Swedish population with a majority of women in the sample (24). The observed sex difference in the present study would be in line with several reports on genotype and sex interactions (39,40) as well as sex differences related to sleep (10,41) and the relation between sleep problems and onset on alcohol use (8). A possible explanation would imply the role of hormones. The *PER2* gene also participates in behavioural processes that are independent of the suprachiasmatic nucleus (SCN) activity, such as alcohol preference, although it is not yet clear in which areas of the brain these extra-SCN rhythms occur, but it has been shown that they can be influenced by hormonal changes (42).

The present study has to be considered in view of its limitations and strengths. A limitation of the first part of this study is that the assessment data on alcohol consumption and sleep problems were from self-reports. There are studies supporting the validity of self-reports in samples of secondary school adolescents (43,44). Nevertheless, the validity of self-reports can always be questioned, and there is reason to believe that in adolescent populations personal interviews outperform questionnaires (45). However, such studies are much more time-consuming and expensive. The three AUDIT-C questions used have been shown to provide a valid measure of hazardous alcohol use, covering frequency of drinking, quantity, and frequency of heavy drinking (30,31). Additionally, other polymorphisms in the *PER2* gene could have been taken into consideration. However, according to Spanagel et al. (25), the SNP 10870 was the one of importance in relation to alcohol consumption. A further limitation could be the lack of details about the sleep problems: if they are due to alcohol consumption and/or if they are not present during long alcohol withdrawal periods. Moreover, with regard to the second part of the study, the results in the severely alcoholic women sample should be considered preliminary and interpreted with caution, since we could only observe a trend for an over-representation of the G allele. However, the control group used was derived from a large population-representative sample of females, which genetically does represent a Swedish population. Additionally, the control group includes both healthy and non-healthy female individuals, wherefore the comparison with the sample of alcoholic females could result in a reduced expected effect size. One strength of the first part of the study is the large number of individuals in the adolescent population. Another strength is the number of questions regarding sleep problems. The 18 multiple-choice items inquire about frequency of sleep disturbances and subjective sleep quality. Furthermore, the procedure with complementary statistical approaches can help to eliminate sources of artefacts in interaction tests. Since in the univariate GLM analyses the AUDIT-C index was used as a quantitative variable, both the model including non-drinkers and the model excluding non-drinkers have been tried. In all cases the GLM and the non-parametric analyses showed a significant interaction effect, thus these similar results give further support for the observed findings. Finally, this study might be of interest for prevention programmes, because the fact that some of those adolescents might develop alcoholism or other psychiatric disorders in adulthood cannot be ignored.

In conclusion, our results have corroborated two new hypotheses that call for further replications. The first is that the *PER2* SNP 10870 polymorphism together with sleep problems is associated with alcohol consumption in a population of adolescent boys. The second is a potential sex difference with regard to the association between the *PER2* 10870 polymorphism and alcoholism.
